# Axonal Transport Deficits in Parkinson’s Disease: Insights from Neurotoxin, Genetic, and Sporadic Models

**DOI:** 10.3390/brainsci16050525

**Published:** 2026-05-14

**Authors:** Xiaobo Wang, Zhaohui Liu, Wanli W. Smith

**Affiliations:** 1 Department of Anatomy and Embryology, School of Basic Medical Sciences, Hangzhou Normal University, Hangzhou 311121, China; 2 Department of Human Anatomy and Embryology, School of Medicine, Northwest University, Xi’an 710069, China; 3 Department of Psychiatry and Behavioral Sciences, Johns Hopkins University School of Medicine, Baltimore, MD 21287, USA

**Keywords:** axonal transport, Parkinson’s disease, α-synuclein, neurotoxins, genetics

## Abstract

Parkinson’s disease (PD) is a prevalent neurodegenerative disorder, characterized by the loss of dopaminergic neurons in the substantia nigra pars compacta and the accumulation of Lewy bodies. Over recent decades, various cellular mechanisms underlying PD have been elucidated, including autophagy, mitochondrial dysfunction, neuroinflammation, and axonal transport. Among them, axonal transport plays a critical role in maintaining the dynamic homeostasis of proteins, membrane-bound organelles, and cellular metabolism within neurons. Unfortunately, a comprehensive overview of axonal transport in PD remains absent. In this review, we synthesized the current literature on axonal transport in PD, leveraging neurotoxic and genetic models to explore the causes and consequences of axonal transport alterations in PD. Through this summary, we aim to deepen our understanding of PD pathogenesis and provide potential therapeutic targets for intervention.

## 1. Introduction

Parkinson’s disease (PD) is a progressive, age-dependent movement disorder that afflicts approximately 1% of individuals over 60 years and 4–5% of those over 85 [1]. Pathologically, PD is characterized by the loss of dopaminergic neurons in the substantia nigra pars compacta and the formation of Lewy bodies, which are cytoplasmic inclusions of α-synuclein (α-syn) aggregates in neurons [2]. Clinical manifestations include bradykinesia, rigidity, postural instability, and resting tremors [2]. While sporadic PD accounts for ~90% of cases, a minority are inherited [3]. Risk factors include exposure to pesticides, alcohol, methamphetamine, and traumatic brain injury [4]. Accordingly, neurotoxin-based PD models, such as those induced by 6-hydroxydopamine (6-OHDA), 1-methyl-4-phenyl-1,2,3,6-tetrahydropyridine (MPTP), paraquat, and rotenone, are widely used in pathological and therapeutic research [5]. Mutations in PD-linked genes, including *SNCA*, *LRRK2*, *PRKN*, and *PINK1*, are implicated in familial forms of the disease [6]. Beyond environmental and genetic factors, diverse pathogenic mechanisms contribute to PD neurodegeneration, including axonal transport [7], inflammatory processes [8] and metabolic dysregulation [9]. The results of functional studies on these genes and their interactions with external risk factors provide extensive insights into the molecular mechanisms of PD pathology.

Axonal transport is a fundamental cellular process essential for maintaining the complex structure and homeostasis of proteins, organelles, and mRNAs in neurons [10]. It is driven by motor proteins, kinesin and dynein, which navigate along polarized microtubules. This process harnesses ATP hydrolysis to fuel intracellular trafficking. Microtubules, key components of the cytoskeleton, are distributed throughout the cell and are assembled through the head-to-tail polymerization of α- and β-tubulin heterodimers. These heterodimers form protofilaments, and thirteen protofilaments further assemble into a hollow tube, named a microtubule [11]. The minus-end (−) of the microtubule is a stable end rich in α-tubulin; in comparison, the plus-end (+) is the dynamic end containing β-tubulin [11]. This polarized architecture enables bidirectional movement of motor proteins in axons, organized in a “plus-end out” principle [11,12]. Axonal transport is indispensable for sustaining healthy neurons, which are highly polarized cells. Motor proteins traverse either away from (anterograde) or toward (retrograde) the cell body along microtubules, regulating mitochondrial energy balance, autophagic–lysosomal degradation, and intracellular transport [13,14]. Disruption of neuronal transport leads to energy imbalance, protein aggregation, neurite injury, and ultimately cell death [13,14]. Axonal transport impairment is recognized as a major contributor to mutant α-syn toxicity and dopaminergic neurodegeneration in PD [15].

In this narrative review, we summarize current knowledge on axonal transport impairment underlying PD pathogenesis, aiming to highlight the role of neuronal transport in neuronal survival and degeneration. The primary goals of this study are to synthesize existing evidence on transport deficits in PD, highlight mechanistic links between transport dysfunction and PD-related pathologies, and to guide future mechanistic and therapeutic studies targeting transport deficits.

## 2. Motor Proteins Involved in Axonal Transport

### 2.1. Kinesin

Kinesin-1 (also referred to as conventional kinesin) mediates anterograde axonal transport toward the microtubule plus-end, with a velocity of approximately 0.5–1 µm/s in cells [16]. Native kinesin holoenzyme comprises two kinesin heavy chains (KHC, 110–130 kD) and two kinesin light chains (KLC, 60–70 kD) [17]. The N-terminal motor domains of KHC bind to microtubules and harness energy from ATP hydrolysis to power movement along the track. KLC subunits are responsible for cargo binding or the regulation of KHC activity. The findings of other studies have demonstrated that kinesin-1 interacts with both tau and α-syn [18]. Despite extensive research, no cell-permeable pharmacological compounds have been developed to selectively inhibit kinesin-1. KIF5A, KIF5B, and KIF5C represent three members of the kinesin-1 family of microtubule-associated motor proteins [19]. KIF5A is structurally organized into three distinct domains: a conserved motor (head) domain spanning amino acids 1–370, a stalk (coiled-coil) domain covering amino acids 371 to 906, and a tail domain from residues 907–1032 [17]. While KIF5B is ubiquitously expressed, KIF5A and KIF5C exhibit predominant expression in neuronal cells. Functionally, KIF5A mediates the axonal transport of diverse cargoes, including synaptic vesicles, mitochondria, lysosomes, and various small protein molecules.

Hereditary spastic paraplegia (HSP) subtype SPG10 is an autosomal dominant disorder caused by mutations in the motor domain of the *KIF5A* gene in humans. SPG10-linked mutations in the motor domain significantly weakened gross cargo flux in vitro [20]. Clinically, SPG10 manifests as a combination of spastic paraplegia, cognitive impairment, peripheral neuropathy, Parkinsonism, or epilepsy. A key pathological hallmark is length-dependent axonal degeneration of the corticospinal tract, which preferentially damages the longest axons connecting to lower motor neurons controlling leg muscles, while the relatively shorter axons innervating arm muscles remain largely unaffected. *KIF5A*-knockout (KO) mice exhibit decreased maximum and average velocity of both bidirectional mitochondria and slowed synaptic transmission in cultured motor neurons, thereby leading to neuronal damage. Of particular note, 75% of constitutive *KIF5A*-KO mice die within 3 weeks, with the remaining mice surviving for up to 3 months, displaying abnormal neurofilament accumulation [21].

### 2.2. Dynein and Dynactin

Dynein mediates retrograde axonal transport toward the microtubule minus-end, opposite to kinesin. Dynein was first identified by Gibbons and Rowe in axonemes [19,22]. The dynein heavy chain (DHC) is a large, conserved protein of approximately 500 kDa, comprising an N-terminal tail domain, a linker domain, and six tandem AAA (ATPases associated with diverse cellular activities) repeat domains at the C-terminus [19]. These AAA domains (1–6) form a hexameric ring that constitutes the dynein head. The microtubule-binding domain is located not on the motor ring but at the tip of a stalk projecting between AAA4 and AAA5. The AAA1 domain primarily provides ATP for motor function, whereas nucleotide binding to AAA3 and AAA4 allosterically modulates dynein activity. The dynein complex is a heavy chain homodimer formed via the N-terminal dimerization domain, with the intermediate and light intermediate chains binding directly to the heavy chain tail and the three types of light chains (LCs) attaching indirectly through the N-terminus of the intermediate chains [23]. Dynein suppression may hamper autophagosome fusion with lysosomes, promoting α-syn-containing aggresome formation [24]. No cytoplasmic dynein mutations have been found to directly contribute to axonal transport impairment in human diseases. However, dynein has been shown to interact with α-syn both in vitro and in vivo [18].

Moreover, dynein requires dynactin, a 1 MDa complex composed of 23 subunits. The main structure of dynactin comprises three major domains: the Arp1 filament, the shoulder-sidearm complex, and the pointed-end complex [25]. The p150Glued dimer, located at the shoulder–sidearm complex, binds to both dynein and microtubules. The pointed-end complex and Arp1 filament promote cargo-binding interactions [26]. Knockdown of *DCTN* in zebrafish recapitulates certain PD features, including decreased dopaminergic neurons and synuclein aggregation through disruption of synuclein autophagosome transport [27].

### 2.3. Myosin

Although myosin is recognized for its role in muscle contraction, it also transports cargos in neurons along filamentous actin, moving toward either the barbed (+) or pointed (−) end [28]. Myosin comprises 18 classes, with different classes presenting distinct structures and functions driven by ATP hydrolysis [29]. In general, myosin consists of three domains: the head domain binds to filaments and hydrolyzes ATP, the neck domain serves as a linker, and the tail domain interacts with cargo. The involvement of myosin in axonal transport has been reported in two studies [30,31]. However, limited evidence is available to explain how myosin regulates axonal transport in PD. Only a few proteomic analyses have suggested that there is a link between myosin and PD or dopamine neurons in the brain [32,33].

## 3. Axonal Transport in Neurotoxin-Induced PD Models

**Figure 1:**
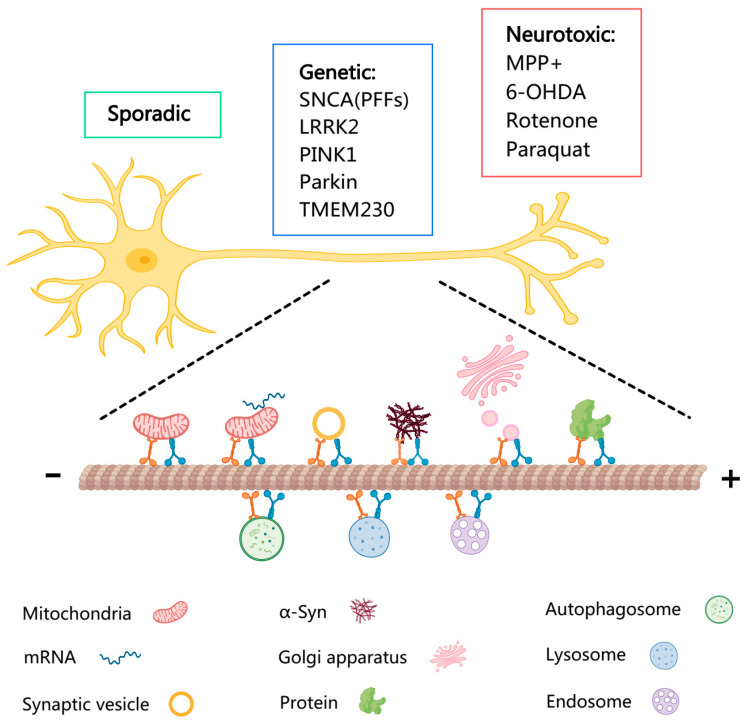
Landscape of axonal transport with a variety of cargos in PD.

Mitochondrial dysfunction in neurons is posited as one of the major mechanisms responsible for dopaminergic neuron degeneration in PD. The metabolite of MPTP, 1-methyl-4-phenylpyridinium (MPP^+^), is known to target mitochondria and produce reactive oxygen species, which trigger neuronal apoptosis directly or indirectly. A similar mechanism is observed in 6-OHDA-, rotenone-, and paraquat-induced PD models. Importantly, mitochondrial dysfunction has recently been highlighted as a vital contributor to neurodegenerative processes in other disorders that clinically resemble PD, such as multiple system atrophy (MSA) and progressive supranuclear palsy (PSP) [34,35]. This emerging evidence further underscores the potential role of mitochondrial impairment as a common pathogenic mechanism across related neurodegenerative conditions, extending beyond PD. An overview of axonal transport with a variety of cargos is presented in Figure 1.

### 3.1. MPP^+^-Induced Models

Initial studies on MPP^+^-induced alterations in axonal transport utilized isolated squid axoplasm [36]. Treatment with 45 μM MPP^+^ enhanced dynein-mediated retrograde transport and inhibited kinesin-1-mediated anterograde transport of membrane-bound organelles, an effect mediated through activation of caspase and protein kinase C [36]. The reduction in synaptic vesicle numbers at presynaptic compartments following transport impairment suggests an early loss of synaptic terminals [36]. Exposure of differentiated PC12 cells to 45 μM MPP^+^ increased retrograde velocity while reducing the anterograde velocity of mitochondria, leading to mitochondrial accumulation within axonal swellings [37]. These findings underscore the vital function of microtubules in MPP^+^-induced intracellular transport disruption and mitochondrial damage [37]. However, dynein levels paradoxically increased after 1 mM MPP^+^ treatment in PC12 cells [24]. Exposure to 1 μM MPP^+^ resulted in a rapid decline in mitochondrial movement within mouse dopaminergic axons, decelerating anterograde transport while accelerating retrograde transport of mobile mitochondria [38]. This modest increase in retrograde transport may contribute to dysregulated mitochondrial trafficking.

Notably, MPP^+^ specifically affected mitochondrial transport without altering synaptic vesicle movements [38]. Treatment with 0.5 mM MPP^+^ increased mitochondrial movement in the retrograde direction while reducing it in the anterograde direction in larval zebrafish [39]. Interestingly, no changes in viability, motor function, or dopaminergic neuron number were observed in zebrafish following MPP^+^ treatment, indicating that axonal transport alterations represent an early event preceding other pathological abnormalities [39]. Furthermore, a higher concentration of MPP^+^ (1 mM) completely abolished overall mitochondrial trafficking [39].

### 3.2. 6-OHDA-Induced Models

Treatment with 60 μM 6-OHDA compromises mitochondrial motility without affecting velocity and induces mitochondrial depolarization in both dopaminergic and non-dopaminergic axons [40]. The mitochondrial transport deficits caused by 6-OHDA can be rescued by reactive oxygen species scavengers. Unlike MPP^+^, 6-OHDA also decreases the number of motile synaptic vesicles in dopaminergic axons [40]. Autophagosome formation, microtubule damage, and subsequent cell death occur sequentially following transport impairment [40]. Thus, by destroying the axonal transport system, 6-OHDA dampens the movement of key cellular cargoes, including mitochondria and synaptic vesicles.

### 3.3. Rotenone-Induced Models

In a chronic rotenone model of rat cortical neurons, which may more closely resemble neurodegenerative diseases than acute models, the authors demonstrate that early alterations in multiple mitochondrial dynamic processes, including fission, fusion, transport, and growth, are coordinately modulated to maintain mitochondrial homeostasis [41]. This process likely contributes to the loss of neuronal processes. Rotenone treatment increases the velocity of extremely small, fast-moving mitochondria in axons, particularly in the retrograde direction [41], which may be the result of downregulated dynein expression [24].

### 3.4. Paraquat-Induced Models

Primary neurons from mice treated with paraquat display mitochondrial dysfunction and bidirectional mitochondrial transport defects owing to KIF5A decline, representing a potential mechanism for paraquat-induced motor deficits and dopaminergic neuronal damage in mice [42].

## 4. Axonal Transport in Genetic PD Models

### 4.1. SNCA

α-Syn is a major component of Lewy bodies. The A30P and A53T mutations in *SNCA*, in addition to *SNCA* gene multiplication, are established causes of autosomal dominant PD. However, *SNCA* pathology is not unique to this disorder. It is the defining feature of a broader group of neurodegenerative diseases known as synucleinopathies, which include dementia with Lewy bodies (DLB) and multiple system atrophy (MSA). These disorders exhibit significant clinical and pathological overlaps with PD in their early stages. For instance, DLB shares cognitive fluctuations and Parkinsonism with PD dementia, whereas MSA presents with atypical Parkinsonism accompanied by autonomic dysfunction and cerebellar ataxia. However, distinct boundaries exist: MSA is characterized by glial cytoplasmic inclusions rather than neuronal Lewy bodies, and DLB features early and prominent visual hallucinations.

#### 4.1.1. α-Syn Protein Transport

Accumulating evidence indicates that impaired neuronal transport of α-syn contributes to Lewy body formation and subsequent neurodegeneration. The authors of early studies classified axonal transport into fast and slow components based on moving rates: fast component (FC, 100–400 mm/day), slow component a (SCa, 0.1–2 mm/day), and slow component b (SCb, 2–10 mm/day). Fast transport primarily involves membrane-bound vesicles and organelles, whereas slow transport mainly carries cytosolic proteins and cytoskeletal components [43]. In the rat optic nerve, α-syn transport is mediated bidirectionally by FC (~25%), in addition to being mediated by SCa (~15%) and SCb in the anterograde direction, with SCb predominating (~60%) [44].

In the fast axonal transport within the rat optic nerve, the A30P mutation abrogates α-syn binding to FC vesicles through the N-terminal repeat segment, which is involved in disease-associated α-syn accumulation [45]. In transgenic mice expressing human SNCA or familial PD-linked mutations (A30P and A53T), α-syn in dorsal root ganglion (DRG) axons is exclusively transported within the SCa/b fraction in wild-type (WT) mice but remains unaffected in mutant strains [46].

However, axonal transport of α-syn in A53T mice progressively slows with aging, resulting in α-syn accumulation within axons and cell bodies [46]. This process may involve elevated kinesin-1 levels [47]. In cultured hippocampal neurons, Roy et al. demonstrated that α-syn, a component of the SCb fraction, moves bidirectionally at approximately 1 μm/s with a 57% anterograde bias. This transport mechanism depends on microtubules rather than actin filaments [48,49]. Current evidence indicates that α-syn indirectly interacts with the anterograde motor kinesin-1 and the retrograde motor dynein to facilitate axonal transport [18,24,50], moving at velocities comparable to those of kinesin-1 and dynein in neurites [51].

In one study, the authors reported that in an AAV2-mediated rat model with A53T overexpression in the striatum, anterograde motor protein levels decreased, whereas retrograde motor protein levels increased [52]. This dysregulation may lead to cargo accumulation in cell bodies, although contradictory changes in dynein have also been observed in A53T transgenic mice [53]. Subsequent alterations in axonal transport, synaptic transmission, cytoskeletal proteins, and neuroinflammatory responses collectively contribute to α-syn-mediated neuronal death [52]. Notably, behavioral assessments were not performed in the A53T rat model.

Additionally, the A30P mutation reduces the traveled distance of α-syn in rat cortical neurons, potentially contributing to α-syn accumulation proximal to the cell body [54]. The authors of another study reported that the A30P mutation perturbs α-syn localization at presynaptic terminals through its lipid-binding N-terminal domain, although the transport moving rate remains unaffected [55]. Intriguingly, the A53T mutation suppresses both the velocity and traveled distance of α-syn yet exhibits no phenotype of α-syn accumulation or aberrant presynaptic localization [54,55]. Furthermore, *SNCA* mutations, including S129E, A30P, and E46K, demonstrate slower transport rates of α-syn in both directions, accompanied by abnormal somatic accumulations in DRG neurons [51]. These findings emphasize the critical importance of microtubule stabilization in α-syn transport.

#### 4.1.2. Transportation of Membrane or Non-Membrane Vesicles

Overexpression of *SNCA*-WT, A30P, and A53T variants in rat primary midbrain neurons compromises the mean velocity, percentage of moving vesicles, and the number of synaptic vesicle speed changes [15], combined with alterations in autophagy, which likely contribute to neuritic morphological damage and axonal degeneration [15]. Excess α-syn or A53T leads to α-syn accumulation that disrupts synaptic protein transport, resulting in synaptic morphological abnormalities and larval locomotion defects. The non-amyloidogenic component region (amino acids 71–82) of α-syn is essential for axonal transport [56]. However, the trafficking of α-syn remains undetected, with only motor protein alterations being reported.

Degradative lysosomes derived from the soma can be transported to distal axons, where they accelerate local α-syn degradation in neurons [57]. Notably, the *SNCA*-A53T mutation impairs the axonal transport of degradative lysosomes, resulting in α-syn accumulation in distal axons [57]. In another study involving the use of human-derived neurons overexpressing empty vectors, *SNCA*-WT, A30P, and A53T to investigate the pathogenesis of PD, normal control neurons displayed higher mitochondrial anterograde flux compared to retrograde flux, and this asymmetry was lost in α-syn variants [58]. The axonal transport alterations were most prominent in the A53T mutant. Taking A53T as an example, the imbalanced mitochondrial distribution can induce mitochondrial fragmentation by disrupting the interaction between the α-syn N-terminal domain and the outer mitochondrial membrane [58]. Moreover, mitochondrial transport defects have been observed in transgenic *SNCA*-A53T zebrafish [59]. Of particular note, the A53T mutation combined with agrochemical exposure triggers deficits in mitochondrial anterograde transport, whereas the mutation alone does not exert this effect. Furthermore, mitochondrial distribution remains undisturbed in A30P rat cortical neurons [54], which may be attributed to the interruption of interactions between KIF5B and nitrated microtubules in human pluripotent stem cells [60].

Overexpression of α-syn impairs retrograde axonal transport of BDNF (brain-derived neurotrophic factor) in transgenic mouse cortical neurons, as α-syn interacts with the dynein intermediate chain and induces endocytic dysfunction through upregulation of Rab5 and Rab7 [61]. Evidence suggests that a deficiency in BDNF supply may cause PD. Knockdown of α-syn affected dynein movement in anterograde directions in DRG neurons, which is hypothesized to be due to the function of α-syn in transportable microtubule polymerization and assembly [51]. Dynein-dependent retrograde transport is also hindered, such as that of lysosomes [51].

#### 4.1.3. Influence of α-syn Oligomers and Fibrils on Transport

Lewy bodies primarily consist of α-syn aggregates, which progress from oligomers to fibrils and accumulate within the cytoplasm. In human induced pluripotent stem cells (hiPSCs) derived from PD patients harboring α-syn gene duplications, oligomeric α-syn was found to bidirectionally disrupt mitochondrial axonal transport while promoting α-syn accumulation [62]. Subsequent studies in hiPSCs carrying oligomer-prone α-syn mutants (E46K and E57K) revealed that α-syn oligomerization impaired anterograde mitochondrial transport, accompanied by the subcellular redistribution of Mitochondrial Rho (Miro1), KLC1 and Tau, leading to energy deficits and ultimately synapse loss [62].

α-Syn preformed fibrils (PFFs) boost the formation of immobile α-syn inclusions in primary hippocampal neurons by recruiting mobile α-syn-positive vesicles [63]. α-Syn aggregates compromise the axonal transport of Rab7 and TrkB-labeled endosomes, in addition to LC3-labeled autophagosomes, without affecting mitochondria and synaptophysin transport. Furthermore, endosome accumulation, morphological abnormalities in endosomes, and defective fusion of autophagosomes with late endosomes/lysosomes were also observed [63]. These findings highlight the mechanisms underlying abnormal protein aggregation and neurodegeneration.

α-SynN103 and tauN368, generated through asparagine endopeptidase cleavage of α-syn and tau, were detected in the brains of PD patients. Preformed α-synN103 and tauN368 fibrils disrupt mitochondrial anterograde transport and endosomal retrograde transport by interrupting the binding between motor proteins and cargo [64]. Dislocated mitochondria and failed neurotrophic factor internalization likely account for neuronal death, with the AMPK and p38/MAPK signaling pathways implicated in this process [64]. α-Syn oligomers, induced by the *SNCA*-E57K mutation, inhibit kinesin–microtubule motility, exacerbating dopaminergic neurite morphology in vivo [65].

Rozan Vroman et al. further corroborated that PFF-induced α-syn propagation in the retrograde direction is more efficient than anterograde propagation, utilizing a novel in vitro microfluidic platform. This approach provides a powerful tool for investigating PD mechanisms and developing treatments aimed at inhibiting α-syn spread [66]. In a similar vein, zebrafish have emerged as an innovative model for studying α-syn aggregation [59].

### 4.2. LRRK2

Functional leucine-rich repeat kinase 2 (LRRK2) modulates substantial intracellular events and neuronal outgrowth essential for neuronal health [67,68]. Emerging evidence indicates that disease-linked *LRRK2* mutants disrupt neural transport, underscoring their pathogenic impact.

#### 4.2.1. *LRRK2* Mutation in the Kinase Domain

The *LRRK2*-G2019S mutation, the most common pathogenic variant in autosomal dominant PD, enhances LRRK2 kinase activity. Researchers have provided evidence that this mutation specifically impairs autophagosomal cargo degradation and perturbs α-syn localization at presynaptic terminals by compromising axonal transport in primary neurons [69,70]. Rab29-mediated LRRK2 hyperactivation phosphorylates RAB proteins and recruits the motor adaptor JIP4 to autophagosomal membranes, thereby disrupting autophagosomal axonal transport, which is associated with failed autophagosome acidification [69]. Furthermore, inhibition of LRRK2 kinase activity restores α-syn anterograde transport to presynaptic terminals, facilitating functional α-syn operation [70]. The authors of future studies should further elucidate the impact of axonal transport in *LRRK2*-G2019S mutation-mediated α-syn aggregation.

#### 4.2.2. LRRK2 Mutation in the ROC-COR Domain

Beyond its kinase domain, LRRK2 contains a Ras of Complex (ROC) GTPase domain and the C-terminal of ROC (COR) domain. Mutations within the ROC-COR domain, including R1441C, R1441H, and Y1699C, are linked to damaged transport of mitochondria, lysosomes, autophagosomes, and synaptic vesicles along axons.

*LRRK2*-R1441C and *LRRK2*-Y1699C mutations disrupt bidirectional mitochondrial transport in Drosophila axons, resulting in locomotor deficits through reduced microtubule acetylation [71]. Notably, enhancing α-tubulin acetylation via deacetylase knockdown or pharmacological inhibition rescues both mitochondrial transport and locomotion in flies harboring *LRRK2* ROC-COR mutations [71]. These findings highlight the therapeutic potential of deacetylase inhibitors in restoring defective axonal transport and mitigating PD symptoms. Our group further demonstrated that *LRRK2*-R1441C expression compromises mitochondrial and lysosomal transport bidirectionally in SH-SY5Y neurites, suggesting that inhibited organelle movement represents an early event in neuronal injury [72]. Intriguingly, the G2019S mutant does not affect mitochondrial or LAMP1-positive vesicle transport in cultured neurons [73]. Additionally, LRRK2 GTP-binding inhibitors of compound 68 and FX2149 not only ameliorate mitochondrial and lysosomal transport defects but also protect against neurodegeneration induced by *LRRK2*-R1441C [72]. Therefore, timely intervention to prevent axonal transport impairment may rescue neuronal loss.

Another *LRRK2* mutation, R1441H, which exhibits higher kinase hyperactivity than G2019S, also impairs axonal autophagosome transport more severely [73]. In the presence of R1441H, stationary autophagosomes increase in number; in comparison, motile autophagosomes present more frequent pauses and prolonged pause durations. A pathogenic “tug-of-war” model has been proposed, suggesting that regulatory imbalance between LRRK2-hyperphosphorylated RABs and GTPase ARF6 leads to dysregulated dynein and kinesin function in opposite directions—a mechanism that also applies to G2019S under the influence of JIP4 [73]. Furthermore, R1441H destroys anterograde axonal transport of synaptic vesicle precursors through RAB3A phosphorylation, resulting in synaptic vesicle accumulation in neuronal soma [74]. This altered synaptic homeostasis is implicated in non-motor symptoms of PD.

#### 4.2.3. *LRRK2* Expression

Overexpression of *Lrrk* (the Drosophila ortholog of human *LRRK2*) inhibits dendrite overgrowth by reducing anterograde transport of Golgi outposts, a mechanism involving Lrrk kinase activity that disrupts the interaction between the golgin Lava lamp and the dynein heavy chain Dhc [75]. However, the *LRRK2*-G2019S mutation leads to a pronounced increase in the retrograde transport of Golgi outposts [75], which is associated with dendrite degeneration. Lrrk2 knockout promotes the accumulation of lysosome-related organelles in neuronal distal regions of Drosophila by increasing Arl8-positive vesicle number in anterograde transport, which may provide a mechanistic basis for the accumulation of α-syn [76].

### 4.3. PINK1 and PRKN

Two autosomal recessive forms of early-onset PD are caused by mutations in *PINK1*, a serine/threonine kinase, and Parkin, an E3 ubiquitin ligase encoded by *PRKN*. Both genes are essential for maintaining mitochondrial function, structure, and dynamics.

#### 4.3.1. *PINK1*

PINK1 acts upstream of Parkin to regulate mitochondrial movement in the axons of both mice and Drosophila models [77]. In this process, PINK1 phosphorylates and degrades Miro in a Parkin-dependent manner, a mechanism triggered by mitochondrial depolarization [77]. Miro is a mitochondrial outer membrane GTPase that attaches kinesin to the mitochondrial surface, thereby facilitating axonal mitochondrial transport [78]. Based on these findings, the authors propose that the PINK1/Parkin pathway may quarantine damaged mitochondria for clearance, thus controlling mitochondrial quality by arresting mitochondrial movement. The findings of another study provided further evidence that knockdown of Miro or PINK1/Parkin-mediated degradation of Miro enhances mitophagy in HeLa cells [79]. In this study, the authors found that *PINK1* overexpression decreases mitochondrial flux and velocity in both directions, whereas *PINK1* knockdown increases anterograde mitochondrial trafficking in larval motor neurons. PINK1 activation or Miro loss improves perinuclear clustering of damaged mitochondria for autophagic clearance and prevents mitochondrial accumulation at motor neuron terminals. However, PINK1-induced changes in mitochondrial length do not correlate with mitochondrial mobility [79]. Recently, the authors of a novel study revealed that PINK1 transcripts can be co-transported with mitochondria to distal regions of neurons for local translation, enabling the removal of damaged mitochondria in a manner dependent on the mitochondrial outer membrane protein SYNJ2BP and the RNA-binding protein SYNJ2a [80]. Consistent with the results of previous studies on axonal transport, active PINK1 impedes the motility of PINK1 mRNA, combined with mitochondria [79].

#### 4.3.2. *PRKN*

Regarding *PRKN*’s role in the regulation of mitochondrial transport, Sung et al. found that in Drosophila larval motor neurons, loss of *PRKN* attenuates mitochondrial flux in both directions, particularly retrograde [81]. This finding is due to the reduction in mitochondrial density in axons but not mitochondrial velocity, morphology, or metabolic state. However, *PRKN* deficiency causes abnormal mitochondrial morphology in the cell body, indicating that *PRKN* protects neurons from dysfunctional mitochondria by maintaining normal somatic mitochondrial dynamics and allowing healthy mitochondrial entry into the axons [81]. In addition to mitochondria, Parkin is also involved in mediating K63-linked polyubiquitination of PD-linked DJ-1 misfolded proteins and promotes the transportation of misfolded proteins to aggresomes in fibroblasts [82]. Mechanistically, polyubiquitinated misfolded DJ-1 binds to the dynein motor via adaptor protein histone deacetylase 6 (HDAC6). Nevertheless, axonal transport dynamics of misfolded DJ-1 have yet to be assessed in the presence or absence of Parkin.

### 4.4. TMEM230

*TMEM230* has been identified as a risk gene for familial PD [83]. The most toxic mutation of *TMEM230*, *184Wext*5, impedes mitochondrial transport in retrograde directions, thereby inducing neuronal death. Notably, re-expression of *TMEM230* rescues defective retrograde mitochondrial trafficking and potentiates neuronal survival in *TMEM230*-knockdown induced neurons [84].

## 5. Sporadic PD

Cybrid cell lines, generated by fusing platelets from sporadic PD patients or disease-free age-matched controls with mtDNA-depleted SH-SY5Y or NT2 cells, provide a powerful approach to investigate mitochondrial dysfunction in PD pathogenesis. In PD cybrid neuronal cells, both the average velocity and total distance of mitochondrial movement are markedly reduced, accompanied by evident mitochondrial damage [85,86,87]. Two therapeutic strategies have been shown to rescue these abnormal mitochondrial dynamics: low-level near-infrared light therapy (810 nm laser), targeting the mitochondrial electron transport chain, and NAP (davunetide), promoting microtubule network assembly [85,86]. Notably, davunetide was evaluated in patients with PSP—a 4R-tauopathy that clinically overlaps with PD at early stages, yet the phase 2/3 trial of davunetide in PSP did not demonstrate efficacy [88]. Additionally, autophagic vesicle trafficking along microtubules is also impaired in PD cybrid neuronal cells, leading to delayed degradation of α-syn and P62 [87].

Notably, alterations in both anterograde and retrograde axonal transport proteins have been confirmed as early features in human sporadic PD brain tissue, with kinesin defects initiating tyrosine hydroxylase (TH) downregulation in the early stages [89]. The reduction in axonal transport proteins is particularly severe in nigral neurons containing α-syn inclusions. Subunits of cytoplasmic LC are only observed during late stages of PD. This phenomenon observed in the substantia nigra is recapitulated in a rat PD model with AAV-mediated overexpression of human A30P α-syn [89]. Characterization of axonal transport in different PD-related cell models is summarized in Table 1.

## 6. Conclusions

In summary, axonal transport plays a critical role in PD pathogenesis, particularly in the trafficking of α-syn and mitochondria. Both neurotoxin-induced PD models and genetically linked PD models significantly affect axonal transport and alter motor protein expression. The imbalance between anterograde and retrograde axonal transport contributes to the “dying-back” mechanism of neurites in PD, which may precede pathological and behavioral changes during disease development. Nevertheless, whether axonal transport deficits are a primary driver of PD pathogenesis or a secondary consequence of other pathological processes, and the temporal relationship between transport impairment and other pathological events require careful investigation in future studies. The alterations in axonal transport observed in PD are associated with cargo diversity, motor protein changes, and the transport infrastructure, including microtubule stabilization. Characterizing transport deficits is not merely a mechanistic exercise but a necessary step toward understanding disease heterogeneity, identifying patient subgroups, and developing rational, transport-based interventions. Clinical trials targeting microtubule stability, while still in early stages, underline the translational potential of axonal transport. In addition, transport deficits that occur in human iPSC-derived neurons from PD patients and in postmortem brain tissues bridge the gap between cellular mechanisms and human pathology.

Despite pronounced advances in axonal transport research in PD, several challenges must be noted. First, is it possible to develop effective drugs or interventions for PD based on mechanisms that restore aberrant axonal transport? While some authors have proposed specific reagents or strategies targeting this mechanism [38,40,71,72,85,86], whether these treatments can alleviate behavioral or non-motor symptoms in PD patients remains unclear. Second, as genetic causes of PD, VPS35 (vacuolar protein sorting-associated protein 35) is a component of the retromer complex, DJ-1 serves as a chaperone sensitive to oxidative stress, and *GBA* encodes glucocerebrosidase, which mediates lysosomal function. At present, only the inhibition of glucocerebrosidase activity has been shown to have no impact on lysosome trafficking [90]. Whether these genes act individually or synergistically with *SNCA*, *LRRK2*, *PINK1*, and *PRKN* to regulate the axonal transport of diverse cargoes and protect neurons warrants further investigation. Third, axonal transport is a complex and delicate process involving numerous proteins beyond kinesin, dynein, and dynactin, such as Hook1 and PI31 [91,92]. A cluster of LRRK2 interactors has also been predicted through computational analysis of the LRRK2 interactome to regulate cell transport or localization [93]. It remains unclear as to whether these proteins affect axonal transport in PD. Finally, clinical translation of axonal transport biology is hampered by the lack of validated biomarkers. Without such tools, it remains impossible to determine whether a failed trial reflects true drug inefficacy, suboptimal target engagement, inappropriate dosing, or enrollment of patients without meaningful transport deficits. Nevertheless, rescuing abnormal axonal transport remains a promising therapeutic approach for PD if these challenges can be systematically investigated and effectively addressed.

While the present review has synthesized current evidence linking axonal transport deficits to clinical features of PD, future research is needed regarding subtype-specific differences in axonal transport dysfunction to explain clinical heterogeneity and the role of axonal transport in prodromal symptom generation to illuminate the earliest stages of disease evolution. Addressing these gaps will not only refine our mechanistic understanding of PD but may also open new avenues for subtype-specific and early-stage therapeutic interventions.

## Figures and Tables

**Table 1:** Summary of axonal transport changes in PD models.

PD Model	Cell Type	Affected Particle	Trafficking Change	Potential Mechanism	Effect	Reference
**Neurotoxin**						
MPP^+^	Squid axoplasm	Membrane-bound organelles	AT ↓ RT ↑	Activation of caspase and PKCδ	Synaptic vesicle reduction	[36]
	PC12 cells	Mitochondria	AT ↓ RT ↑	MT overstabilization, reorientation, and reduced MT dynamics	Mitochondrial accumulation in axons	[37]
	Mouse DA neurons	Mitochondria	AT ↓ RT ↑	——	Mitochondrial depolarization	[38]
	Zebrafish DA neurons	Mitochondria	AT ↓ RT ↑	——	No pathological or behavioral change	[39]
6-OHDA	Mouse DA and non-DA neurons	Mitochondria	Motile numbers ↓	Oxidative stress	Autophagosome formation, MT disruption, and cell death	[40]
	Mouse DA neurons	Synaptic vesicles	Motile numbers ↓	——		
Rotenone	Rat primary cortical neurons	Fast and small mitochondria	AT ↑ RT ↑	Dynein downregulation	Mitochondrial homeostasis impairment	[41]
Paraquat	Mice primary midbrain neurons	Mitochondria	AT ↓ RT ↓	KIF5A downregulation	Mice motor deficits and dopaminergic neuronal damage	[42]
**Genetics**						
***SNCA***						
A30P, A53T	Rat primary midbrain neurons	Synaptic vesicles	Mean velocity ↓ Motile numbers ↓ Number of speed changes ↓	——	Damaged neurite outgrowth and ramification	[15]
S129E, A30P, E46K	Rat DRG neurons	α-syn	AT ↓ RT ↓	——	Abnormal accumulations of α-syn	[51]
Knockdown	Rat DRG neurons	Lysosomes	RT ↓	α-syn binds to MT and regulates its polymerization	——	
		Protein dynein	Motile numbers ↓			
A53T	Rat striatum	——	AT protein ↓ RT protein ↑	——	Cargo accumulation in the cell body	[52]
A30P	Rat primary cortical neurons	α-syn	Traveled distance ↓	——	α-syn accumulation close to the cell body	[54]
A53T			Velocity ↓	——	No phenotype	
A30P	Rat primary hippocampal neurons	α-syn	——	α-syn lipid-binding N-terminal domain	Disruption of α-syn localization	[55]
A53T			Mean velocity ↓	——	No phenotype	
A53T, excess α-syn	Neuromuscular junctions of larvae	Synaptic proteins	Transport impairment	Non-amyloidal component region of α-syn	Abnormal synaptic morphology	[56]
A53T	Mouse DRG neurons	Degradative lysosomes	Transport impairment	——	α-syn accumulation in distal axons	[57]
A30P, A53T	Human-derived neurons	Mitochondria	Transport impairment	Disruption of α-syn binding with the outer mitochondrial membrane	Mitochondrial fragmentation	[58]
A53T+ rotenone/paraquat	Human iPSC neurons	Mitochondria	Time portion of AT ↓	Nitrated α-tubulin inhibits the binding of α-syn and KIF5B to MT	——	[60]
Expression of GFP-α-syn	Transgenic mouse cortical neurons	Protein BDNF	RT velocity ↓ Paused periods ↑	α-syn interacted with the dynein intermediate chain	Endocytic dysfunction	[61]
E46K, E57K	Human iPSC neurons	Mitochondria	AT numbers ↓	Miro1, KLC1, and Tau relocation	Energy deficits and synapse loss	[62]
*SNCA* duplication	Human iPSC neurons	Mitochondria	AT ↓ RT ↓	——	Accumulation of α-syn	
PFF	Mice primary hippocampal neurons	α-syn	AT number ↓	——	α-syn inclusion formation	[63]
		Late endosomes	RT ↓	——	Abnormal endosome morphology	
		Autophagosomes	Motile numbers ↓ AT velocity ↑ RT velocity ↑	——	Abnormal autophagosome fusion with lysosomes	
PFF (α-synN103 +tauN368)	Mice primary cortical neurons	Mitochondria	AT ↓	Disrupt binding to motor proteins	Mitochondria dislocation	[64]
		Endosomes	RT ↓	Disrupt binding to motor proteins	Neurotrophic factor endocytosis	
***LRRK2***						
G2019S	Mice primary cortical neurons	Autophagosomes	Transport impairment	LRRK2 kinase activity on Rabs and motor adaptor JIP4 recruitment	Failure of autophagosomal cargo degradation	[69]
G2019S	Mice primary cortical neurons	α-syn	AT ↓	——	Failure of α-syn presynaptic localization	[70]
R1441C, Y1699C	Rat cortical neurons, drosophila motor neurons	Mitochondria	Motile numbers ↓	Decreased microtubule acetylation	Locomotor deficits	[71]
R1441C	SH-SY5Y cells	Mitochondria	Motile numbers ↓ RT distance ↓	——	Neuritic injury	[72]
		Lysosomes	Motile numbers ↓ RT distance ↓	——		
R1441H	Human iPSCs	Autophagosomes	Transport impairment	LRRK2 kinase activity on Rabs and ARF6 regulation	Autophagy inhibition	[73]
R1441H	Human iPSCs	Synaptic vesicles	AT ↓	Rab3a phosphorylation	Limit synaptic vesicles to soma	[74]
*Lrrk* overexpression	Drosophila neurons	Golgi outposts	AT ↓ Stationary Golgi outposts ↑	Lrrk kinase activity inhibits the interaction between Lva5 and DHC	Suppression of dendrite arborization	[75]
*Lrrk* knockout	Drosophila neurons	Arl8 protein	AT ↑	——	Lysosome accumulation	[76]
***PINK1&PRKN***						
*PINK1* and *PRKN* expression	Rat hippocampal neurons	Mitochondria	Time proportion of AT and RT particles ↓	Degrades Miro in motor/adaptor complex and releases kinesin from mitochondria	Clearence of damaged mitochondria	[77]
*PINK1* expression	Drosophila larval motor neurons	Mitochondria	AT and RT ↓	——	Abnormal mitochondrial distribution	[79]
*PINK1* knockdown	Drosophila larval motor neurons	Mitochondria	AT ↑	——		
*PINK1* expression	Rat and mouse hippocampal neurons	Mitochondria with PINK1 mRNA	AT and RT ↓	Cotransport with mitochondria	Mitophagy in the distal end of the axon	[80]
*PRKN* deletion	Drosophila larval motor neurons	Mitochondria	AT and RT ↓	Reduced mitochondrial density	Abnormal mitochondrial morphology	[81]
*PRKN* deletion	Fibroblasts	DJ-1 protein	——	Binding to dynein via HDAC6	Aggresome formation	[82]
***TMEM230***						
*TMEM230* knockdown	Mouse hippocampal neurons	Mitochondria	RT ↓	Low TMEM230 expression	Neuronal death	[84]
**Sporadic PD**						
Sporadic	Cybrid cell lines	Mitochondria	Average velocity ↓ Total traveled distance ↓	——	Mitochondria dysfunction	[85]
	Cybrid cell lines	Autophagic vesicles	Motile numbers ↓ Average velocity ↓	——	α-syn accumulation	[87]
	Human brain tissue	——	AT and RT protein ↓	KHC and LC reduction	TH decrease	[89]

Anterograde transport (AT); Retrograde transport (RT); Microtubule (MT); Upward arrow (increase); Downward arrow (decrease).

## Data Availability

No new data were created or analyzed in this study.

## Abbreviations

The following abbreviations are used in this manuscript:

6-OHDA	6-hydroxydopamine
α-syn	α-synuclein
AAA	ATPases associated with diverse cellular activities
AD	Alzheimer’s disease
ALS	Amyotrophic lateral sclerosis
BDNF	brain-derived neurotrophic factor
DCTN	dynactin
DHC	dynein heavy chain
DLB	dementia with Lewy bodies
DRG	dorsal root ganglion
FC	fast component
GBA	glucocerebrosidase
hiPSCs	human induced pluripotent stem cells
HSP	Hereditary spastic paraplegia
KHC	kinesin heavy chains
KIF5A	kinesin family member 5A
KLC	kinesin light chains
LC	dynein light chain
Miro	Mitochondrial Rho
MPP^+^	1-methyl-4-phenylpyridinium
MPTP	1-methyl-4-phenyl-1,2,3,6-tetrahydropyridine
MSA	Multiple system atrophy
NAP	davunetide
PFFs	preformed fibrils
PINK1	PTEN-induced kinase 1
PRKN	parkin RBR E3 ubiquitin protein ligase
PSP	Progressive supranuclear palsy
SC	slow component
SPG10	Spastic paraplegia-10
TH	tyrosine hydroxylase
VPS35	vacuolar protein sorting-associated protein 35
